# Pandemic treaty should include provisions for palliative care

**DOI:** 10.2471/BLT.23.289957

**Published:** 2023-06-01

**Authors:** Katherine Pettus, Stephen Connor, Julia Downing, Joan Marston

**Affiliations:** a International Association for Hospice and Palliative Care, 5535 Memorial Drive, Suite F-509, Houston, Texas 77007, United States of America.; b Worldwide Hospice and Palliative Care Alliance, London, England.; c International Children’s Palliative Care Network, Bristol, England.; d Palliative Care in Humanitarian Aid Situations and Emergencies (PallCHASE), Scotland.

The coronavirus disease 2019 (COVID-19) pandemic stress-tested health system capacities worldwide to provide both essential and emergency services. Essential services include palliative care, which before the pandemic, the World Health Organization (WHO) estimated was available to 14% of people who need it.^1^ Most health systems failed the COVID-19 stress test as they shifted all available resources to treat COVID-19 patients. Health institutions that had personal protective equipment scrambled to provide it to frontline workers, many of whom were diverted to intensive care from what were deemed non-essential services, including palliative care. Those institutions that de-prioritized or halted diagnostics, treatments and palliative care for non-COVID-19 patients with chronic conditions and noncommunicable diseases are still struggling to manage the backlogs.^2^

Even as higher-income countries gradually sorted out therapeutics and vaccines for their population, the literature reveals that both COVID-19 and non-COVID-19 patients in countries of all income levels had insufficient palliative care support.^3^^–^^7^ The existing global inequity in access to services and medicines worsened the suffering of patients, families and health workers, who otherwise could have received emotional and spiritual support. In response, clinical and academic experts across the world have called for the explicit inclusion of palliative care in future pandemic planning.^8^ However, those responsible for drafting and negotiating the WHO pandemic preparedness and response treaty have not heeded that call. Neither the preamble nor the operational paragraphs of the zero draft mention the words suffering, rehabilitation or palliative care.^9^

As health systems and hospitals diverted the palliative care workers they had available to emergency and intensive care units when the pandemic started, their counterparts in those and other clinical specialties had to learn palliative care skills in real-time when they could no longer save patients’ lives. Clinicians who had never had to conduct end-of-life conversations or relieve terminal breathlessness improvised under pressure, often experiencing moral injury when they were unable to provide effective help. Overwhelmed emergency units either struggled or managed brilliantly under the circumstances, often while confronting supply chain breakdowns that triggered stock-outs of internationally controlled essential medicines for the relief of pain and dyspnea.^10^

Lessons learned from those many months prompted the four international palliative care organizations to participate in the Intergovernmental Negotiating Body deliberations as relevant stakeholders. We prepared and distributed an advocacy note^11^ asking governments to explicitly include palliative care in the new treaty before the next pandemic breaks out.

Behind each COVID-19-related death was a person whose suffering could have been relieved by palliative care, whether medical countermeasures could have saved their life or not.^12^ A failure to acknowledge the suffering the pandemic caused is an offence to clinical and global health ethics, both of which are based on by principles of beneficence and justice. Integrated into health systems from the community through to tertiary levels, palliative care supports population health and builds system-level resilience. Member States do not need to wait for the treaty to strengthen health workforces and invest in palliative care.

We suggest that governments apply to the World Bank Pandemic Fund to support their investment in palliative care, and to train on the following skill sets: working with ethically acceptable decision algorithms to ration treatment when resources are scarce; effective symptom management for severe pain and respiratory distress, including prescribing of internationally controlled essential medicines such as morphine and midazolam; alternative delivery methods for palliative care services such as telemedicine; psychosocial and spiritual support for patients, health workers and caregivers; and bereavement care. Governments can partner with national, regional and global palliative care associations to do so. Many unprepared or underprepared health-care workers who cared for dying patients during and after the pandemic experienced emotional distress that could be prevented or mitigated. Such preparation aligns with Article 12 of the zero draft, and is relevant to other health emergencies and humanitarian crises.

The advocacy note respectfully suggests where treaty negotiators can include the words suffering, palliative care and rehabilitation in the text, following agreed United Nations language from the 2018 Declaration of Astana on Primary Health Care, and World Health Assembly resolutions. Many global and allied professional organizations, including the World Organization of Family Doctors, have endorsed the note, as have 54 regional and national palliative care associations at the time of writing.
